# CELF2 regulates the species-specific alternative splicing of *TREM2*

**DOI:** 10.1038/s41598-020-75057-x

**Published:** 2020-10-22

**Authors:** Motoaki Yanaizu, Chika Washizu, Nobuyuki Nukina, Jun-ichi Satoh, Yoshihiro Kino

**Affiliations:** 1 Department of Bioinformatics and Molecular Neuropathology, Meiji Pharmaceutical University, 2-522-1, Noshio, Kiyose-shi, Tokyo 204-8588 Japan; 2 Laboratory for Structural Neuropathology, RIKEN Brain Science Institute, 2-1 Hirosawa, Wako-shi, Saitama 351-0043 Japan; 3 Laboratory of Structural Neuropathology, Doshisha University Graduate School of Brain Science, Kyoto, 610-0394 Japan

**Keywords:** Biochemistry, Molecular biology, Neuroscience, Diseases, Neurology, Risk factors

## Abstract

Genetic variations of *TREM2* have been implicated as a risk factor of Alzheimer’s disease (AD). Recent studies suggest that the loss of TREM2 function compromises microglial responses to the accumulation of amyloid beta. Previously, we found that exon 3 of TREM2 is an alternative exon whose skipping leads to a reduction in full-length TREM2 protein by inducing nonsense-mediated mRNA decay. Here, we aimed to identify factors regulating TREM2 splicing. Using a panel of RNA-binding proteins, we found that exon 3 skipping of TREM2 was promoted by two paralogous proteins, CELF1 and CELF2, which were both linked previously with risk loci of AD. Although the overexpression of both CELF1 and CELF2 enhanced exon 3 skipping, only CELF2 reduced the expression of full-length TREM2 protein. Notably, the *TREM2* ortholog in the green monkey, but not in the mouse, showed alternative splicing of exon 3 like human *TREM2*. Similarly, splicing regulation of exon 3 by CELF1/2 was found to be common to humans and monkeys. Using chimeric minigenes of human and mouse *TREM2*, we mapped a CELF-responsive sequence within intron 3 of human *TREM2*. Collectively, our results revealed a novel regulatory factor of TREM2 expression and highlighted a species-dependent difference of its regulation.

## Introduction

Alzheimer’s disease (AD) is the leading cause of dementia and is a progressive neurodegenerative disorder characterized by the pathological accumulation of amyloid beta (Aβ) plaques and neurofibrillary tangles^1^. To date, genetic studies have identified at least 29 genes whose variants are associated with the susceptibility to AD^2^. Some risk genes are expressed in microglia^3,4^, which are tissue-resident macrophages of the central nervous system and are involved in the development of brain circuits and maintenance of the neuronal environment through synaptic pruning, phagocytosis, and cytokine release^5–7^. Reflecting their diverse roles, microglia have been implicated in various aspects of the pathophysiology of AD, with findings suggesting both protective and harmful effects^8^. TREM2 (triggering receptor expressed on myeloid cells 2) is an immune receptor highly expressed on microglia^9,10^. Hypomorphic *TREM2* variants, such as R47H, confer higher risks of developing AD^11,12^. Moreover, loss-of-function mutations of *TREM2* cause Nasu–Hakola disease (NHD), which is characterized by early-onset dementia with leukoencephalopathy and bone cysts^13^. *TREM2* mutations have also been found in frontotemporal dementia without bone involvement^14,15^. Thus, the functions of TREM2 are essential for maintaining brain functions during aging. TREM2 binds to several ligands, including anionic and zwitterionic lipids, APOE, and Aβ^16–18^. The variants associated with AD and NHD impair the binding of TREM2 to its ligands or the production of the mature protein^17,19^. While microglia surround Aβ deposits in AD brains, reflecting the phagocytosis of Aβ and/or the formation of barriers against Aβ, *TREM2*-deficient microglia do not cluster around Aβ deposits, leading to an increased burden of Aβ and worsened axonal dystrophy^16,20^. In contrast, the increased gene dosage of *TREM2* was shown to ameliorate the pathology and memory deficits of AD model mice^21^. Microglia undergo phenotypic changes in response to the environment in the brain. In addition to classically known pro-inflammatory M1 microglia and anti-inflammatory M2 microglia, recent single-cell RNA sequencing studies have revealed specific subtypes of microglia in response to various conditions, including aging and neurodegenerative diseases^22–25^. Disease-associated microglia (DAM) are characterized by a unique transcriptional signature, including the upregulation of TREM2 and APOE^23,24^. In mice, Trem2 is an essential factor for the transition of homeostatic microglia to DAM, as *Trem2*-deficient mice failed to develop mature DAM^23^. These findings suggest that TREM2 plays a key role in the AD-associated microglial functions. In addition to DAM, TREM2 is also important for the transition of monocytes into lipid-associated macrophages emerging in obese tissues^26^, which may prevent metabolic abnormalities, suggesting a broader importance of TREM2 in disease-associated conditions.

Previously, we identified a regulatory mechanism associated with TREM2 expression through an attempt to develop a therapeutic strategy for a *TREM2* mutation (c.482 + 2T>C) that causes NHD^27^. This splice-site mutation results in the skipping of TREM2 exon 3, which produces a premature termination codon (PTC) on exon 4 that, in turn, induces the destabilization of mRNA through nonsense-mediated mRNA decay (NMD). Interestingly, we noticed that exon 3 skipping also occurs in wild-type TREM2, but is hardly detectable due to degradation by NMD. Thus, TREM2 protein expression is altered through the alternative splicing of exon 3. In humans, most protein-coding genes are under the control of alternative splicing events, such as the inclusion or skipping of exons, switching of splice sites, retention of introns, and mutually exclusive splicing^28,29^. These forms of alternative splicing are achieved by coordination between the spliceosome and RNA-binding proteins (RBPs)^30^. Therefore, it is likely that the alternative splicing of TREM2 exon 3 is regulated by certain RBPs, which might serve as key factors of microglial activity by regulating TREM2.

To identify the splicing regulators of exon 3, we screened 34 RBPs using a TREM2 minigene and identified CELF1 and CELF2 as candidates. CELF1 and CELF2 belong to the CELF family and have been shown to regulate RNA processing, including mRNA stability, splicing, and translation^31–34^. Interestingly, both *CELF1* and *CELF2* have been suggested to be genes conferring susceptibility to AD in genome-wide association studies (GWAS)^35,36^. CELF2 downregulates TREM2 protein expression by inducing exon 3 skipping. Interestingly, the alternative splicing of exon 3 is species-specific and is not conserved in mouse Trem2. In addition, a chimeric minigene assay and RNA immunoprecipitation (RIP) suggested that intron 3 of human TREM2 mediates the effect of CELF2. Taken together, our results provide a novel molecular link between TREM2 and CELF proteins and reveal a species-specific difference of TREM2 that may be important for the disease modeling of AD and other neurological diseases involving microglia and TREM2.

## Results

### Screening of RBPs for the regulators of TREM2 exon 3 splicing

**Figure 1:**
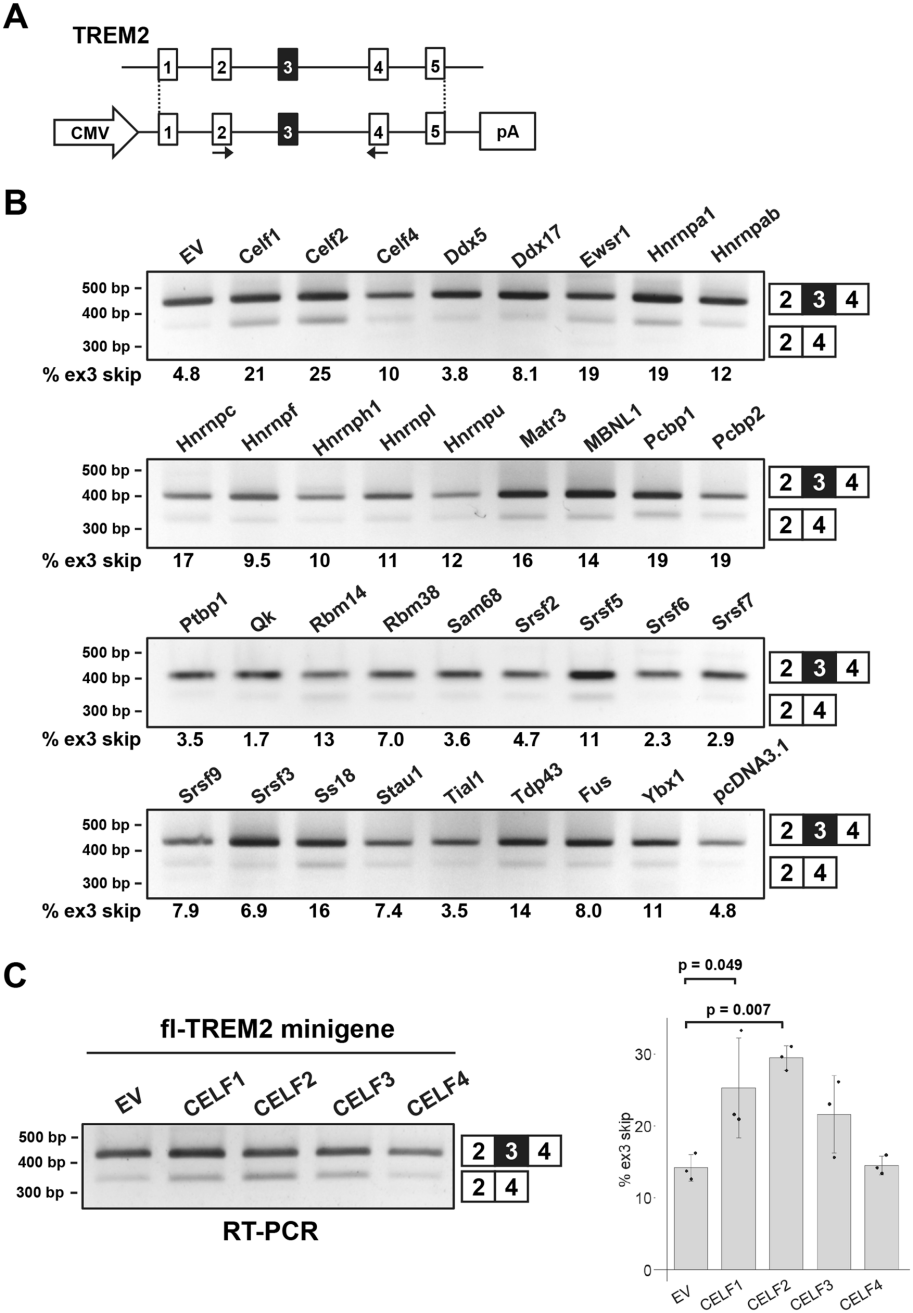
Screening of RNA-binding proteins (RBPs) that regulate the alternative splicing of TREM2 exon 3. (**A**) Schematic diagram of the fl-TREM2 minigene. The arrows indicate the primer set to detect the splicing pattern of exon 3. (**B**) Results of the splicing assay using the fl-TREM2 minigene and a panel of RBPs. RBPs were expressed as a fusion with EGFP. Splice products were detected by RT-PCR using agarose gels. The proportion of exon 3 skipping is indicated at the bottom of each lane. EGFP (EV) and pcDNA3.1 were used as controls. (**C**) The effect of human CELF proteins (CELF1/2/3/4) on the splicing of TREM2 exon 3. The fl-TREM2 minigene was co-transfected with EGFP-fused CELF proteins into HEK cells. Bar chart shows the proportion of exon 3 skipping. Error bars represent SD (n = 3). Tukey’s test was used for statistical evaluation.

To identify the factors regulating the alternative splicing of TREM2 exon 3, we selected 34 RBPs derived from murine cDNA (except for MBNL1 of human origin) and expressed them as EGFP-fused proteins. These RBPs were selected because they are either expressed in microglia or reported to be associated with neurodegenerative diseases (Supple. Table S1). The EGFP-fused RBPs were transfected into HEK cells with a TREM2 minigene covering the genomic region from exon 1 to exon 5 (fl-TREM2 minigene, Fig. 1A). As HEK cells do not express TREM2 at detectable levels^27^, TREM2 mRNA/protein signals detected hereafter are transgene-derived. The splicing pattern of TREM2 exon 3 was determined by reverse transcription-polymerase chain reaction (RT-PCR) (Fig. 1B). We observed that some RBPs either increased or decreased exon 3 inclusion to variable degrees in comparison with that in the negative controls (EGFP and pcDNA3.1). Notably, exon 3 skipping was most strongly promoted by two paralogous proteins, Celf1 and Celf2 (Fig. 1B, Supple. Fig. S1A). We observed less strong skipping of exon 3 by hnRNPA1, Ewsr1, Pcbp1, and Pcbp2 (Fig. 1B). Conversely, Ptbp1 appeared to promote exon 3 inclusion (Fig. 1B). Among these RBPs, we selected Celf1 and Celf2 for further analyses because (1) they altered exon 3 splicing most strongly and (2) both have been implicated as genes conferring susceptibility to AD in GWAS^35,36^. Moreover, CELF1 expression is higher in isolated human microglia than that in unfractionated brain cortex, suggesting that CELF1 expression is enriched in microglia^3^. Similarly, a single-cell atlas indicated that CELF2 is also enriched in microglia among cell types in the human entorhinal cortex (https://adsn.ddnetbio.com/)^37^. We compared human CELF proteins, including CELF3 and CELF4. As with their murine counterparts, human CELF1 and CELF2 induced exon 3 skipping (Fig. 1C). While CELF3 showed a tendency for enhanced exon 3 skipping, CELF4 did not alter exon 3 splicing (Fig. 1C). We also used a minigene (TREM2 ex2–4) covering the genomic region of TREM2 from exon 2 to exon 4, in which NMD is not induced by exon 3 skipping. As expected, CELF1 and CELF2 increased exon 3 skipping of the TREM2 ex2–4 minigene (Supple. Fig. S1B), suggesting that the genomic region of TREM2 exon 2 to exon 4 is sufficient to mediate exon 3 skipping by CELF1 and CELF2. Taking these findings together, the alternative splicing of TREM2 exon 3 is regulated by several RBPs, among which Celf1 and Celf2, as well as their human orthologs, exhibit the strongest effects. The possibility that some human RBPs potently regulate human TREM2 splicing but were missed because their murine orthologs are much less active cannot be ruled out.

### CELF2 downregulates TREM2 protein expression by inducing exon 3 skipping

**Figure 2:**
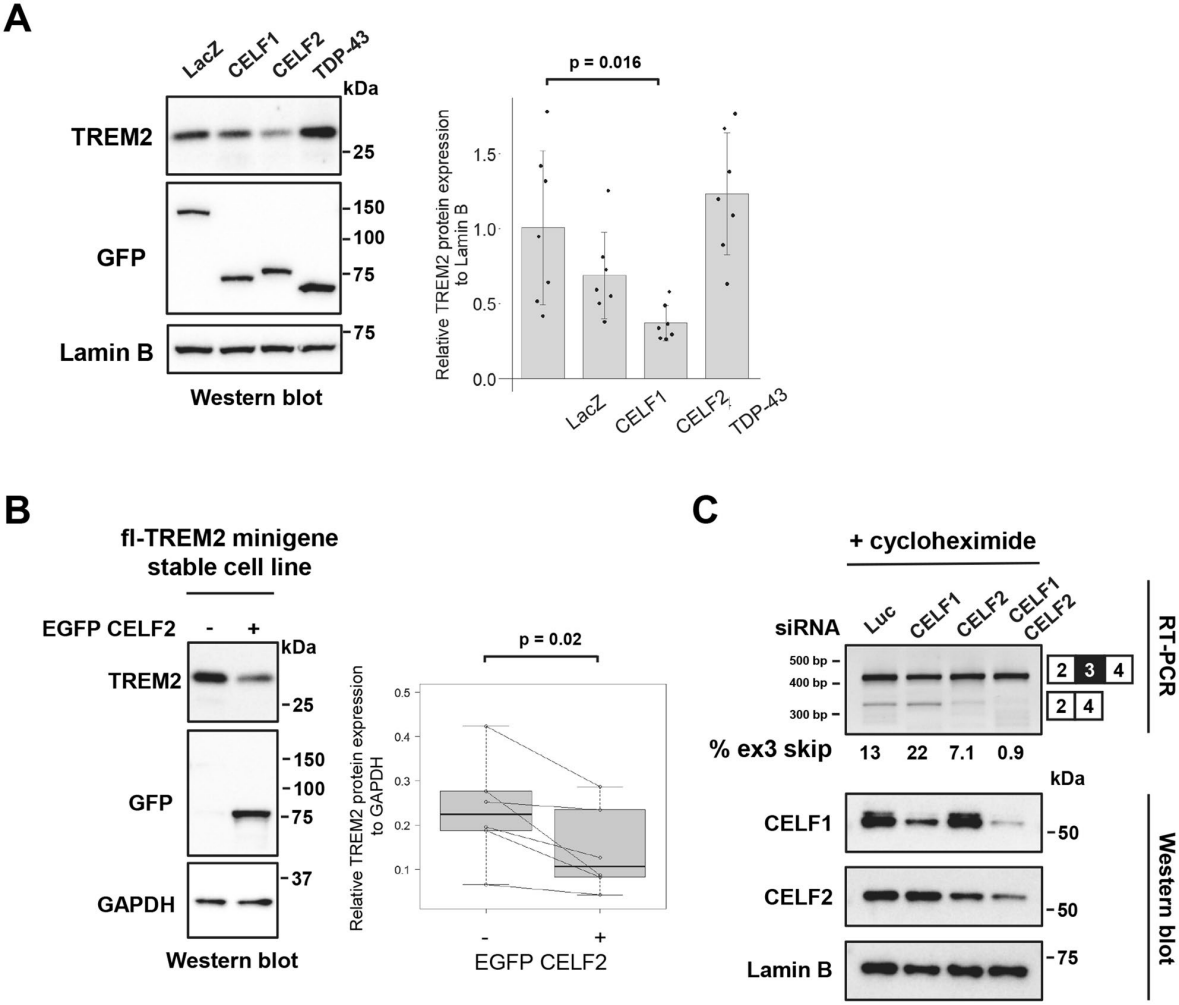
CELF2 promotes exon 3 skipping and decreases the protein expression of TREM2. (**A**) Western blot analysis of the fl-TREM2 minigene co-transfected with EGFP-fused CELF1 or CELF2 into HEK cells. Protein expression was detected using antibodies as indicated. EGFP-LacZ was used as a control. Bar chart shows TREM2 protein levels relative to Lamin B (arbitrary unit). Error bars represent the SD (n = 7). Tukey’s test was used for statistical evaluation. (**B**) HEK cells that stably express the fl-TREM2 minigene were transfected with EGFP-CELF2, and the EGFP-positive cells were separated from the EGFP-negative cells via fluorescence-activated cell sorting. The cells were subjected to western blot, using antibodies as indicated (lest panel). The box plot shows TREM2 protein expression levels relative to GAPDH (arbitrary units). The collected EGFP-positive and EGFP-negative cells were considered as a pair at each sorting, and the two-tailed paired *t* test was used for statistical analysis (n = 6). (**C**) RT-PCR products of TREM2 in THP-1 cells treated with siRNA and cycloheximide (CHX) were resolved by polyacrylamide gels (upper panel). The lower panel shows western blot results of CELF proteins in siRNA-treated THP-1 cells.

As reported previously, TREM2 protein expression is partly regulated by its exon 3 splicing^27^. Therefore, we examined the effect of CELF proteins on the protein level of TREM2. The fl-TREM2 minigene was co-transfected with CELF1 or CELF2 into HEK cells. Western blot analysis revealed the downregulation of the TREM2 protein levels by CELF2 (Fig. 2A). CELF1 also exhibited a tendency for downregulation, but the difference did not reach significance (Fig. 2A). We also tested TDP-43, as this protein recognizes UG-rich sequences, like CELF1 and CELF2^38–42^. However, TDP-43 altered neither the alternative splicing of exon 3 nor TREM2 protein expression (Fig. 2A, Supple. Fig. S2A). TREM2 is expressed in both membrane-bound and soluble protein forms^19^. Membrane-bound, cytoplasmic, and extracellular (culture medium) fractions were prepared from HEK cells, with and without stable TREM2 expression, apart from total cell lysates. When these cells were compared, it was determined that the major TREM2 band in the total cell lysate (~ 28 kDa) corresponded to the membrane-bound TREM2 (Supple. Fig. S2B). We also detected secreted TREM2 in the culture medium that migrated as a faint smear observed at approximately 30–40 kDa (indicated by an asterisk, Supple. Fig. S2B). The membrane-bound TREM2 protein level was downregulated by CELF2 overexpression (Supple. Fig. S2C). We also performed flow cytometry-based western blot analysis using HEK cell line that stably expresses the fl-TREM2 minigene^27^. The cells were transfected with EGFP-fused CELF2 and then sorted according to their EGFP fluorescence. The EGFP-positive cells showed a significant decrease in the TREM2 protein levels compared with the EGFP-negative cells (Fig. 2B). Furthermore, TREM2 immunofluorescence analysis showed that CELF2 overexpression resulted in a decrease in TREM2 expression in the cells stably expressing fl-TREM2 minigene as well as THP-1 cells expressing a detectable level of endogenous TREM2 (Supple. Fig. S3). Next, we examined whether exon 3 skipping is decreased when the expression of CELF1 and/or CELF2 is suppressed. As the spliced product lacking exon 3 is degraded as a target of NMD, it is difficult to detect the splicing pattern of exon 3, especially in THP-1 cells^27^. In the presence of CHX to inhibit NMD, THP-1 cells showed an elevated ratio of exon 3 inclusion when CELF1 and/or CELF2 were suppressed by RNAi (Fig. 2C). Similar splicing patterns were observed in the fl-TREM2 minigene stable cell lines (Supple. Fig. S2D). These results suggested that both CELF1 and CELF2 are involved in the regulation of exon 3 splicing.

### The reduction of TREM2 expression through the skipping of exon 3 by CELF2

**Figure 3:**
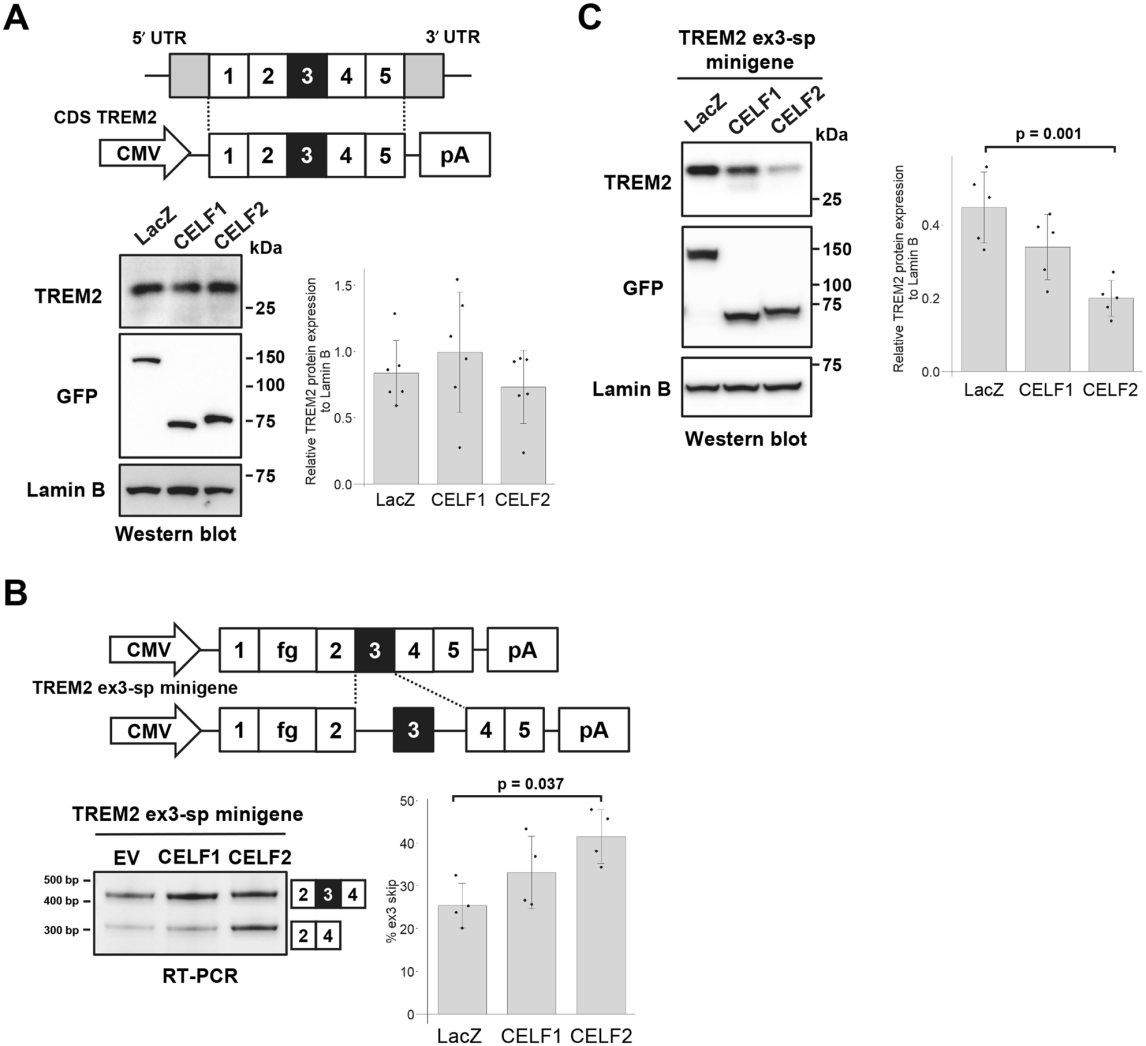
The reduction of TREM2 expression by CELF2 is coupled with exon 3 skipping. (**A**) Schematic diagram of the CDS TREM2 construct (upper panel). Western blot analysis of CDS TREM2 that was co-transfected into HEK cells with EGFP-fused CELF1 or CELF2 (lower left panel). Quantification of TREM2 expression levels (lower right panel). Bar chart shows TREM2 protein levels relative to Lamin B (arbitrary units). Error bars represent the SD (n = 6). No significant alteration of TREM2 protein expression was observed (Tukey’s test). Lamin B was used as a loading control as well as a reference for normalization. (**B**) Schematic diagram of the TREM2 ex3-sp minigene. The fragment of the genomic region of TREM2 intron 2 to intron 4 was inserted into TREM2 cDNA. fg represents the 3xDDDDK tag (upper panel). Splicing assay results of the TREM2 ex3-sp minigene co-expressed with CELF proteins using polyacrylamide gels (lower left panel). Bar chart shows the portion of exon 3 skipping (lower right panel). Error bars represent the SD (n = 4). Tukey’s test was used for statistical evaluation. (**C**) Western blot analysis of TREM2 ex3-sp co-transfected with EGFP-fused CELF1 or CELF2 into HEK cells (left panel). Bar chart shows TREM2 protein levels relative to Lamin B (right panel). Error bars represent the SD (n = 5). Tukey’s test was used for statistical evaluation.

In addition to splicing, CELF1 and CELF2 are known to regulate translation^33,43^. The reduction of the TREM2 protein expression level by CELF2 could be due to the translational regulation by this protein, independently of splicing. To test this, we prepared a TREM2 cDNA construct lacking all introns (CDS TREM2) and transfected it into HEK cells with EGFP-fused CELF1 or CELF2. The TREM2 protein was not decreased by CELF1 or CELF2 (Fig. 3A), demonstrating that the downregulation of TREM2 protein by CELF2 requires introns or their processing.

To further investigate the effect of CELF proteins on the expression of TREM2 protein, we constructed a minigene in which only exon 3 can be spliced (Fig. 3B, referred to as TREM2 ex3-sp minigene). TREM2 ex3-sp showed the facilitation of exon 3 skipping by CELF2 (Fig. 3B). In addition, TREM2 protein was also decreased by CELF2 (Fig. 3C). CELF1 showed a similar tendency; however, the differences in the effects were not significant (Fig. 3B,C). These results indicated that the reduction of TREM2 protein expression was caused by the promotion of exon 3 skipping by CELF2.

### Alternative splicing of TREM2 exon 3 is not conserved in mice

**Figure 4:**
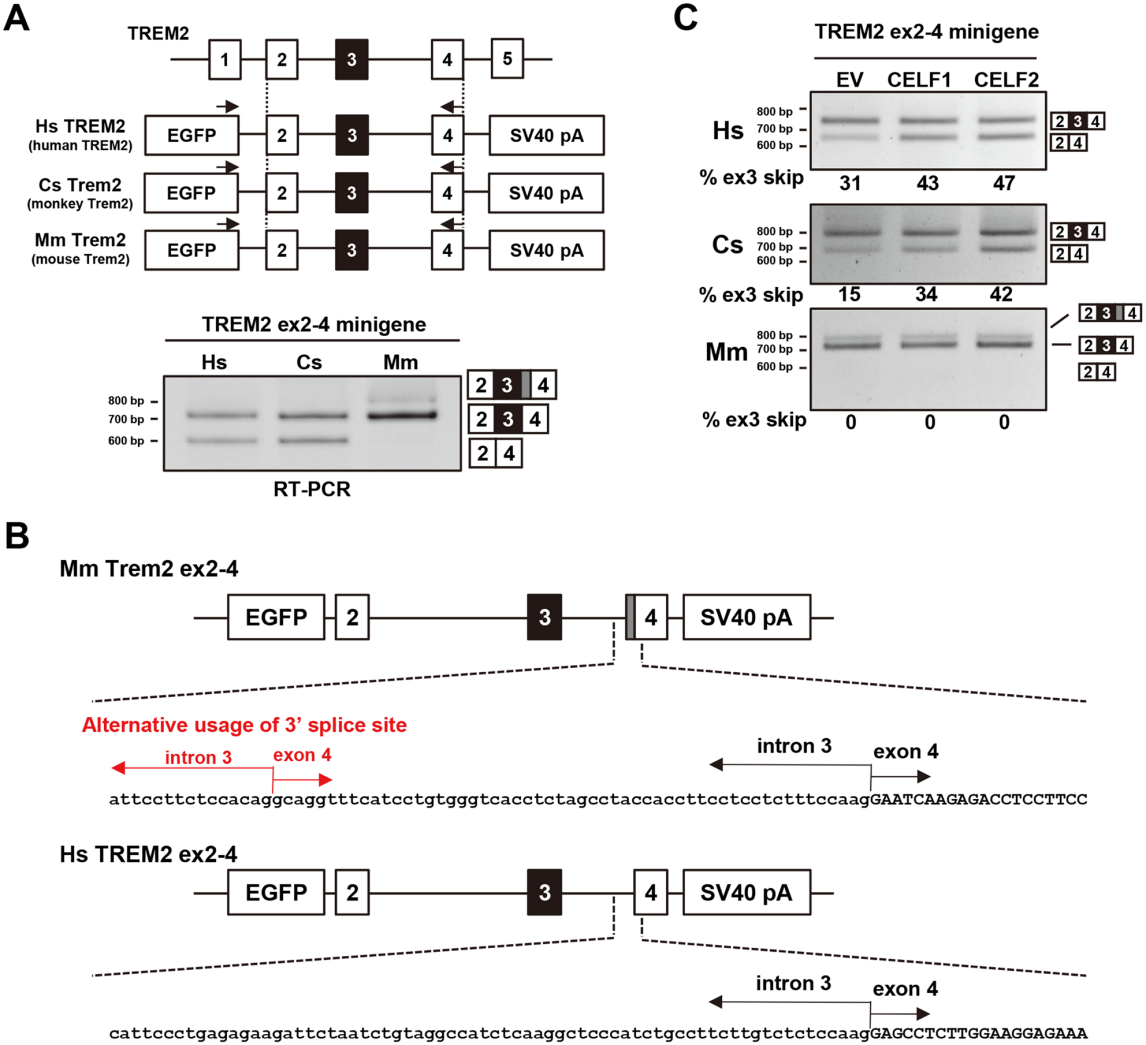
Primate-specific alternative splicing of TREM2 exon 3 is regulated by CELF1 and CELF2. (**A**) Schematic diagram of minigenes covering a genomic region from exon 2 to exon 4 of *Homo sapiens* TREM2 (HsTREM2), *Chlorocebus sabaeus* Trem2 (CsTrem2), and *Mus musculus* Trem2 (MmTrem2). Each fragment was inserted into the pEGFP-C1 vector. The black arrows show the primer sets for RT-PCR to detect the splicing pattern of exon 3 in each species. A species-specific reverse primer was used in this experiment. RT-PCR products were resolved by polyacrylamide gels. (**B**) Schematic diagram of MmTrem2-specific splice products represented by a gray box. These splice products were confirmed by sequencing. (**C**) Each minigene was co-transfected with EGFP-fused cDNA into HEK cells to detect the alteration of exon 3 splicing. RT-PCR products were resolved by agarose gels.

We next investigated the conservation of the alternative splicing of TREM2 exon 3. Two minigenes covering a genomic sequence from TREM2 exon 2 to exon 4 of *Chlorocebus sabaeus* Trem2 (CsTrem2) and *Mus musculus* Trem2 (MmTrem2) were constructed to compare the splicing patterns of exon 3 in these species with that in humans (Fig. 4A). Interestingly, the alternative splicing of TREM2 exon 3 was observed in CsTrem2 like in human TREM2, but not in MmTrem2. No endogenous mouse MmTrem2 exon 3 skipping was observed in RAW264.7 cells even when NMD was inhibited by CHX (Supple. Fig. S4A). This indicated that the alternative splicing of TREM2 exon 3 is primate-specific or at least species-dependent. Additionally, MmTrem2 showed a mouse-specific splice product as a minor band, which was produced by the alternative usage of 3′ splice sites and contained the last 55 nucleotides of intron 3 inserted between exons 3 and 4 (Fig. 4B)^4^. This product was also observed when the splicing of endogenous Trem2 of RAW264.7 was tested (Supple. Fig. S4A).

We then tested the effect of CELF proteins on TREM2 minigenes from different species. RT-PCR analysis indicated that exon 3 skipping was increased by CELF1 or CELF2 in primate TREM2, but not in MmTrem2 (Fig. 4C). Lastly, murine Celf proteins were tested for the splicing of MmTrem2 in mouse-derived cells (Neuro2a). In this combination, again, exon 3 skipping was not detected in the mouse MmTrem2 minigene (Supple. Fig. S4B). There was no major difference in the expression levels of CELF1 and CELF2 among human and mouse cell lines, except for a relatively high abundance of CELF1 in PMA-treated THP-1 (Supple. Fig. S4C). In conclusion, the alternative splicing of exon 3 was conserved between primate species, but not in the mouse, and is regulated by CELF1/2 in the former species.

### TREM2 intron 3 mediates the splicing regulation by CELF2

**Figure 5:**
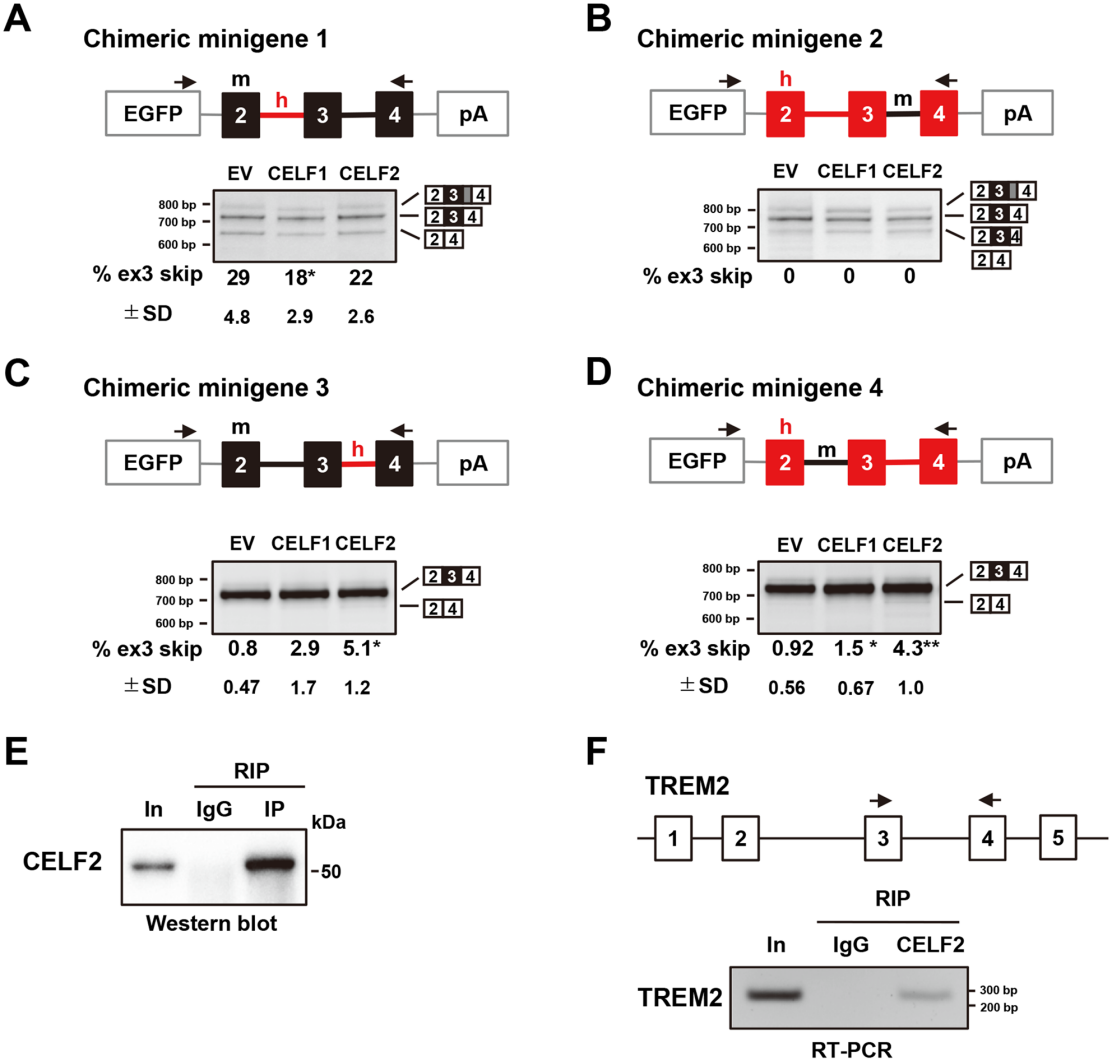
Chimeric minigene assay and RNA immunoprecipitation to map the determinants of the alternative splicing of exon 3. (**A–D**) The results of the chimeric minigene assay. Chimeric minigenes of human and mouse TREM2 were inserted into the pEGFP-C1 vector. The black boxes and lines indicate mouse exons and introns, respectively. The red boxes and lines indicate human exons and introns, respectively. The arrows exhibit the primer set. RT-PCR products were resolved by polyacrylamide gels. Tukey’s test was adopted for statistical tests (n = 4). (**E**) Western blot analysis to confirm the immunoprecipitation of CELF2 using the anti-CELF2 antibody. *In* input fraction. (**F**) RT-PCR analysis of cDNA synthesized from CELF2-immunoprecipitated products. The primer set, indicated by the black arrows, was used to detect the binding of CELF2 to TREM2 RNA.

We reasoned that comparisons of human TREM2 with mouse MmTrem2 can reveal the regulatory mechanisms of the alternative splicing of exon 3. For this purpose, four chimeric minigenes were prepared by combining the mouse MmTrem2 ex2-4 minigene and the human TREM2 ex2-4 minigene (Fig. 5). Chimeric minigene 1, in which intron 2 of MmTrem2 was replaced with that of the human, showed an unexpected decrease in exon 3 skipping by CELF1, but no significant changes by CELF2 (Fig. 5A). These differential effects between CELF1 and CELF2 may underlie the weaker suppressive activity of CELF1 on exon 3 skipping. This result also suggested that human intron 2 alone was not sufficient to mediate the effect of CELF2. Chimeric minigene 2, in which intron 3 of human TREM2 was replaced with that of the mouse, showed a unique pattern, which was not altered by CELF proteins (Fig. 5B). Here, all three bands that were detected contained exons 2, 3, and 4, with some differences in exon 4 splicing. The longest product had an extended exon 4 sequence, as observed for MmTrem2. The shortest product lacked the first 74 nucleotides in exon 4, suggesting that the combination of mouse intron 3 and human exon 4 might activate a cryptic 3′ splice site in human exon 4. Since no exon 3 skipping was observed for this minigene, intron 3 of human TREM2 was essential for exon 3 to be alternatively spliced. Chimeric minigene 3, in which intron 3 of MmTrem2 was replaced with that of the human, showed little exon 3 skipping under basal conditions (Fig. 5C), suggesting that human intron 2 facilitates the alternative splicing of exon 3. Interestingly, small but significant exon 3 skipping was detected when CELF2 was co-expressed (Fig. 5C). Similar results were obtained for chimeric minigene 4, in which intron 2 of human TREM2 was replaced with that of the mouse (Fig. 5D). As both chimeric minigenes 3 and 4 contained human intron 3, these results demonstrate that human intron 3 is essential for both the alternative splicing of exon 3 and its regulation by CELF2. In addition, human intron 2 appeared to predispose exon 3 to be alternatively spliced.

Finally, we investigated the binding of CELF2 to the endogenous TREM2 transcripts using RIP. Western blot results revealed that CELF2 was expressed in THP-1 and was immunoprecipitated using the anti-CELF2 antibody (Fig. 5E). The amplification of the region of TREM2 exons 3–4, including intron 3, was detected by RT-PCR using the CELF2-immunoprecipitated RNA as a template, suggesting the binding of CELF2 and TREM2 pre-mRNA (Fig. 5F). Taking these findings together, the regulation of exon 3 splicing by CELF2 is at least partly mediated by intron 3 of human TREM2, which is physically bound by CELF2.

## Discussion

Our RBP screening identified CELF1 and CELF2 as novel splicing regulators of TREM2 exon 3. CELF1 and CELF2 recognize UG-rich sequences, and UG-rich and UGUU-rich sequences, respectively^39,41,42^. Despite their preferences for similar sequences, we observed a functional difference between CELF1 and CELF2. While both proteins promoted exon 3 skipping, only CELF2 significantly decreased TREM2 protein expression (Figs. 1C, 2A). Furthermore, chimeric minigene 1 showed different responses to these proteins (Fig. 5A). CELF1 overexpression, but not CELF2, induced an additional weak band of TREM2, smaller than the major band, with the TREM2 ex3-sp minigene (Fig. 3C). Although the identity of this band has not been determined yet, it may also suggest some difference between CELF1 and CELF2 regarding TREM2 regulation. Possibly, either CELF protein has additional binding sites on the TREM2 pre-mRNA that are not shared by the other, or CELF1 and CELF2 bind to the same sites, but exert distinct effects by binding to different protein interactors. In previous examples, CELF2 was shown to regulate alternative splicing by interacting with U2 snRNA-associated proteins^44,45^ or with hnRNPC^46^. Further detailed analyses would reveal previously unknown differences between CELF1 and CELF2 and the mechanism by which CELF2 regulates exon 3 splicing.

Several studies have shown that single-nucleotide polymorphisms (SNPs) in both *CELF1* and *CELF2* are associated with AD. An SNP present in the intronic region of *CELF1* was identified as a polymorphism conferring susceptibility to AD^35^. More recently, however, *SPI1* (also known as *PU.1*), which flanks *CELF1*, has been reported to contain more likely causal variants associated with AD^47^. Both of these gene variants function as *cis-*expression quantitative trait loci that modulate the expression levels of nearby genes^35,47,48^. In addition, the SNP rs201119 located within an intron of *CELF2* is a significant risk factor for AD for the carriers of the *APOE* e4 allele^36^, which is the strongest genetic risk factor for AD^49,50^. Although the exact effect of the *CELF2* risk allele is still unknown, the regulatory relationship between CELF2 and TREM2 is intriguing given that TREM2 is also functionally linked with APOE^17^.

Although little has been revealed about splicing regulation in microglia to date, recent findings imply its importance in the context of AD. For example, an SNP of *CD33*, another gene conferring a risk of AD, affects the alternative splicing of its second exon^51,52^, which alters the structure of the ligand recognition region of CD33 and leads to the phagocytic activation of microglia^53,54^. Therefore, splicing regulators of AD-related genes may influence the pathology of AD by altering the function of these genes. Furthermore, *Tdp-43*-deficient microglia promote amyloid uptake^55^. Here, our results highlight CELF2 as a splicing regulator of a microglial hub gene, *TREM2*. It would be interesting to determine whether CELF2 regulates other microglial genes associated with AD in coordination with TREM2.

Increasing attention has been paid to TREM2 since its relevance to AD was discovered. This protein is a potential biomarker and a potential therapeutic target of AD^21^. Membrane-bound TREM2 is cleaved by ADAM10 and secreted as a soluble protein (sTREM2). Higher levels of sTREM2 in the cerebrospinal fluid are associated with reduced cognitive and clinical decline in individuals with mild cognitive impairment or AD^56^. Moreover, supplementation of sTREM2 in the brain of AD model mice ameliorated disease progression^57^. The secreted form of TREM2 is also produced by the alternative splicing of exon 4, which encodes a transmembrane domain^58^. Exon 3 skipping causes a frameshift, resulting in the introduction of a PTC. The resultant isoform lacking exon 3 is degraded by NMD^27^. However, because the efficiency of NMD depends on the cell type or context^59^, a fraction of TREM2 mRNA lacking exon 3 escapes degradation and is subjected to translation, as observed in a patient with a splice-site NHD mutation^60^. When exon 3 is skipped, the transmembrane domain is lost due to frameshifting. In fact, secreted TREM2 was observed in the culture medium when TREM2 cDNA lacking exon 3 was expressed in cultured cells (Supple. Fig. S5). It will be interesting to determine whether NMD activity is altered with aging in microglia.

A species-dependent difference is an important issue in medical research, including that on AD^61^. Here, we have revealed that TREM2 splicing diverges between humans and mice. Although the significance of this finding in the pathophysiology of AD is currently unclear, we speculate that there might be other species-dependent differences in AD-associated genes that are currently unrecognized. In line with this, a functional difference of human and mouse CD33 has been reported recently^62^. These results reinforce the importance of human-derived experimental systems in modeling diseases, such as iPS cells and chimeric mice transplanted with human-derived cells^63,64^, in addition to conventional animal models. Remarkably, although several groups have established *Trem2* knock-in mice harboring the R47H risk variant in the mouse *Trem2* gene^65,66^, this substitution was found to cause aberrant splicing and the subsequent downregulation of mRNA through NMD that do not occur in human TREM2^66^. Regarding exon 3 splicing, R47H substitution in the fl-TREM2 minigene did not alter the splicing pattern (Supple. Fig. S6). Comparative analyses of the expression of microglial genes from various species revealed that microglia show divergence in terms of morphology and gene expression, including the difference between primates and rodents^67^. Our analysis indicated that intron 3 of human TREM2 mediates the repressive effect of CELF2, while intron 3 of mouse Trem2 contains an extra splice site that leads to an elongated exon 4, demonstrating a differential response of TREM2 orthologs attributed by the difference of genomic sequences in these species. A major limitation of this study is that we did not examine the splicing of TREM2 in the microglia of different species.

In summary, our results revealed that CELF2 is a novel regulator of the alternative splicing of TREM2 exon 3. This suggests that CELF2 may affect the function of microglia by regulating the expression level of TREM2. Moreover, we suggest that the alternative splicing of TREM2 exon 3 is primate-specific. This latter finding may be crucial in interpreting the results obtained by using mouse microglia in the context of neurodegenerative diseases involving TREM2.

## Conclusions

We identified CELF1 and CELF2 as modulators of the alternative splicing of TREM2 exon 3. CELF2 reduced the protein level of TREM2 significantly, whereas CELF1 showed a weaker effect on the TREM2 protein expression. Notably, the alternative splicing of TREM2 exon 3 was conserved in the green monkey, but not in the mouse. Moreover, CELF1 and CELF2 increased the skipping of exon 3 in primate TREM2, but not in mouse Trem2. Comparative analyses of human–mouse chimeric minigenes suggested that intron 3 of human TREM2 mediates the effect of CELF2. Our results demonstrate that CELF2 regulates the species-specific splicing of TREM2, providing new insights into the regulation of TREM2 and its species-dependent differences.

## Materials and methods

### Plasmids

The fl-TREM2 minigene and TREM2 ex2–4 minigene were as described previously^27^. DDDDK-tagged TREM2 was engineered by inserting a 3xDDDDK (3xFlag) tag, which was amplified from p3xFLAG-CMV-7.1 (Sigma-Aldrich), downstream of the signal peptide sequence of TREM2 cDNA that had been cloned into pcDNA3.1-hygro (Invitrogen). The TREM2 ex3-sp minigene was prepared by replacing the exon 3 sequence of DDDDK-tagged TREM2 with the genomic sequence covering from intron 2 to intron 3, which had been amplified from the fl-TREM2 minigene. This fragment was inserted into the NheI/NotI site of the pCMV-FRT vector^27^. For the construction of CDS TREM2, the TREM2 cDNA fragment was amplified from the cDNA library of THP-1 cells and digested with NheI and NotI. This fragment was inserted into the NheI/NotI site of pCMV-FRT. To investigate the conservation of alternative splicing of TREM2 exon 3, the following minigenes were prepared: *Chlorocebus sabaeus* Trem2 (Cs Trem2) exon 2 to exon 4 from COS-7 genomic DNA and *Mus musculus* Trem2 (Mm Trem2) exon 2 to exon 4 from Neuro2a genomic DNA. A genomic fragment from TREM2 exon 2 to exon 4 of each species was amplified by PCR and then digested with BclI and SalI. The products were inserted into the BglII/SalI site of the pEGFP-C1 vector (Clontech). For chimeric minigene construction, the genomic regions corresponding to the species were amplified using species-specific primers. Subsequently, fragments were assembled by NEBuilder HiFi DNA Assembly Master Mix (NEW ENGLAND BioLabs), according to the manufacturer’s instructions. These assembled fragments were digested with BclI and SalI, and then inserted into the BglII/SalI site of the pEGFP-C1 vector. To make TREM2 dE3, DDDDK-tagged TREM2 was amplified with using two set of primers: NehI-TREM2-ex1-Fw and TREM2-delE3-Rv and TREM2-delE3-Fw and NotI-TREM2-ex5-Rv. These two fragments were combined into one fragment by PCR and digested with NheI and NotI. The construction of TREM2 dE4 was similar to that of TREM2 dE3. The primers used in this study are listed in Supple. Table S2.

Human CELF constructs, EGFP-MBNL1_42_ and EGFP-Fus (denoted previously as EGFP-Tls), were as described previously^68,69^. Other RBPs were amplified from FANTOM3 cDNA clones^70^ or directly from a mouse cDNA library using a pair of gene-specific primers containing a restriction site. Amplified fragments were inserted into the BglII/SalI sites of pEGFP-C1. The details of the RBP constructs are listed in Supple. Table S3.

### Cell culture

HEK cells stably expressing the fl-TREM2 minigene were established in our previous study^27^. HEK cells (RCB1637, Riken BRC), Neuro2a cells (#CCL-131, ATCC), RAW264.7 cells (#91062702, ECACC), HeLa cells (RCB0007, Riken BRC), and fl-TREM2 stable cell lines were grown in Dulbecco’s modified Eagle’s medium supplemented with 10% fetal bovine serum (FBS) and 1% penicillin/streptomycin (Thermo Fisher Scientific) at 37 °C in 5% CO_2_. THP-1 cells (RCB1189, Riken BRC) were maintained in RPMI medium supplemented with 10% FBS, 1% penicillin/streptomycin, 1 × GultaMAX (Thermo Fisher Scientific), and 2-mercaptoethanol (Wako) at 37 °C in 5% CO_2_. THP-1 cells were treated with 100 µg/ml cycloheximide (CHX; Wako) for 6 h at 37 °C to inhibit NMD.

### Cellular splicing assay

A cellular splicing assay was performed in accordance with that in our previous study^27^. For this splicing assay, HEK cells were seeded on 12-well plates coated with 0.1% v/w gelatin (Wako) on the day before transfection. A total of 0.02 µg of minigene expression vector and 0.48 µg of EGFP-fused protein expression vector were transfected using Lipofectamine 2000 (Thermo Fisher Scientific). To introduce siRNA, THP-1 cells were seeded on 24-well plates and transfected with 20 pmol siRNAs (the sequences of siRNAs are listed in Supple. Table S2) per well by Lipofectamine RNAiMAX (Thermo Fisher Scientific). 72-h after siRNA transfection, THP-1 cells were treated with 100 µg/ml CHX (Wako) for 6 h at 37 °C to inhibit NMD. Total RNA was harvested using the NucleoSpin RNA kit including DNase treatment (TaKaRa). Reverse transcription (RT) was performed using Revertra Ace-α- (TOYOBO) with oligo dT and random hexamers as a primer. The amount of RNA was adjusted to the same volume among the samples. RT-PCR was carried out using the Blend-Taq-plus- (TOYOBO) and primer sets listed in Supple. Table S2. Electrophoresis was performed using agarose gels (Invitrogen and pH Japan) or e-PAGEL polyacrylamide gels (ATTO). The gels were stained with ethidium bromide (Genesee Scientific Corporation). The appropriate number of PCR cycles was determined by sampling at multiple cycles. Gel images were captured by Luminograph III (ATTO) and quantified using CSAnalyzer (ATTO). Original gel images are shown in Supple. Fig. S7.

### SDS-PAGE and western blotting

Western blotting was carried out in accordance with that in our previous study^27^. To detect the secreted protein in the culture medium, 0.5 µg of DDDDK-tagged TREM2 was transfected into HEK cells seeded on 12-well plates. The culture medium was harvested 72-h after transfection. After removing cell pellets by centrifugation, trichloroacetic acid (Wako) at a final concentration of 10% was added. To promote precipitation, sodium deoxycholate (Wako) was added at a final concentration of 10%. The culture medium containing trichloroacetic acid and sodium deoxycholate was incubated at − 80 °C for 1 h. Next, the thawed culture medium was centrifuged at 16,100 × *g* for 15 min to collect the precipitated products. After centrifugation, the supernatant was removed and the precipitated products were washed with cold acetone. This process was repeated twice, after which the precipitated products were dried at 37 °C for 30 min. Finally, the precipitated products were solubilized with SDS sample buffer and boiled. The cell were fractionated as previously described^71^, with two minor modifications: all steps were performed without a phosphatase inhibitor, and we used an EGTA-free membrane protein isolation buffer. The signals were captured by Luminograph III (ATTO) and quantified using CSAnalyzer (ATTO). The antibodies used in this study are listed in Supplementary Table S4. Original gel images are shown in Supple. Fig. S7.

### Immunofluorescence

HEK cells and THP-1 cells were cultured in four- or eight-well chamber slides (WATSON). The cells were fixed with 4% PFA for 30 min at room temperature. Cells were treated with PBS containing 0.1% Triton X-100 for 5 min at room temperature. After blocking with 5% skim milk for 30 min, the cells were incubated with primary antibody overnight at 4 °C. Next, the cells were incubated with Alexa 488- or 568-conjugated secondary antibody (Thermo Fisher Scientific) for 1 h at room temperature. After removing the secondary antibody, the cells were washed with PBS. A mounting medium with DAPI (VECTOR LABORATORIES) was applied to detect nuclei. Fluorescence images were captured by confocal microscopy (LSM710; Carl Zeiss).

### Fluorescence-activated cell sorting

HEK cells that stably express the fl-TREM2 minigene were seeded on gelatin-coated 6-cm dishes the day before transfection. EGFP-fused CELF2 was transfected using Lipofectamine 3000 (Thermo Fisher Scientific), and cells were collected 72-h after transfection for sorting. They were subsequently trypsinized and resuspended in DMEM supplemented with 10% FBS and 1% penicillin/streptomycin. The cells were then passed through a cell strainer to eliminate clumps and debris and sorted using a MA900 cell sorter (SONY) to separate the EGFP-positive cells from the EGFP-negative cells. The instrument parameters were set according to the manufacturer’s instructions. Once sorted, the cells were subjected to SDS-PAGE and western blot.

### RNA immunoprecipitation

THP-1 cells were cultured in a six-well plate and treated with PMA. Cells were fixed with 1% PFA for 10 min at room temperature. For the quenching of PFA, 1.5 M glycine was added at a final concentration of 0.25 M for 5 min at room temperature. Cells were collected and sonicated in RIPA buffer containing 0.1 U/µl RNase inhibitor and 1 × protease inhibitor (Complete; Roche) for 30 s, six times at intervals of 1 min. The cell pellet was fractionated by centrifugation at 16,100 × *g* for 15 min. The supernatant was pre-cleared with Dynabeads protein A (Thermo Fisher Scientific) at 4 °C for 1.5 h. A portion of the pre-cleared lysate was collected as input and the rest was immunoprecipitated using Dynabeads protein A magnetic beads conjugated with anti-CELF2 antibody or rabbit IgG overnight at 4 °C. The beads were washed three times with modified RIPA buffer containing 1 M NaCl and 1 M urea. After washing, the beads and the input fraction were reverse cross-linked by incubation at 70 °C for 1 h in an elution buffer containing 50 mM Tris–HCl (pH 7.4), 1% SDS, 1 mM EDTA, and 50 mM 2-mercaptoethanol. The input and eluted fractions were analyzed by western blotting. RNA was purified by NucleoSpin RNA (TaKaRa) with DNase treatment following phenol/chloroform precipitation. cDNA was synthesized by RT using Revertra Ace-α-. Co-immunoprecipitated RNA was amplified by RT-PCR using the primers listed in Supplementary Table S2.

### Statistical analysis

All graphs were produced using R (version 3.6.1, https://www.r-project.org/). EXCEL Toukei software (ESUMI Co., Ltd.) was used in all cases to conduct statistical analyses. Error bars in all graphs represent standard deviations (SD). The data were analyzed using two-tailed unpaired *t* test for Supple. Figs. S2A and S6. Two-tailed paired *t* test was also used, as shown in Fig. 2B. The remaining data were analyzed using one-way analysis of variance, followed by Tukey’s test. The information on statistical tests is shown in each figure.

## Supplementary information

Supplementary Tables.

Supplementary Figures.

## Data Availability

The datasets generated and/or analyzed during the current study are available from the corresponding author on reasonable request.
