# Supplementary material for: CELF2 regulates the species-specific alternative splicing of TREM2
Source: Sci Rep. 2020 Oct 22;10:17995. doi: 10.1038/s41598-020-75057-x (PMC7582162; doi:10.1038/s41598-020-75057-x)
Supplement: Supplementary file 2 — Supplementary Figures. [file 41598_2020_75057_MOESM2_ESM.pdf]

Supplementary Information

CELF2 regulates the species-specific alternative splicing of TREM2

Motoaki Yanaizu[1], Chika Washizu[2], Nobuyuki Nukina[2,3],
Jun-ichi Satoh[1], Yoshihiro Kino[1,2,*]

[1]Department of Bioinformatics and Molecular Neuropathology,
Meiji Pharmaceutical University, 2-522-1, Noshio, Kiyose-shi,
Tokyo 204-8588, Japan
[2]Laboratory for Structural Neuropathology, RIKEN Brain Science Institute,
2-1 Hirosawa, Wako-shi, Saitama 351-0043, Japan
[3]Laboratory of Structural Neuropathology, Doshisha University
Graduate School of Brain Science, Kyoto 610-0394, Japan

[*]Corresponding author: Tel.: +81-42-495-8679, E-mail: kino@my-pharm.ac.jp

E-mail addresses:
Motoaki Yanaizu: d186902@std.my-pharm.ac.jp
Chika Washizu: chika.eagle.m@gmail.com
Nobuyuki Nukina: nnukina@mail.doshisha.ac.jp
Jun-ichi Satoh: satoj@my-pharm.ac.jp
Yoshihiro Kino: kino@my-pharm.ac.jp

Figure. S1

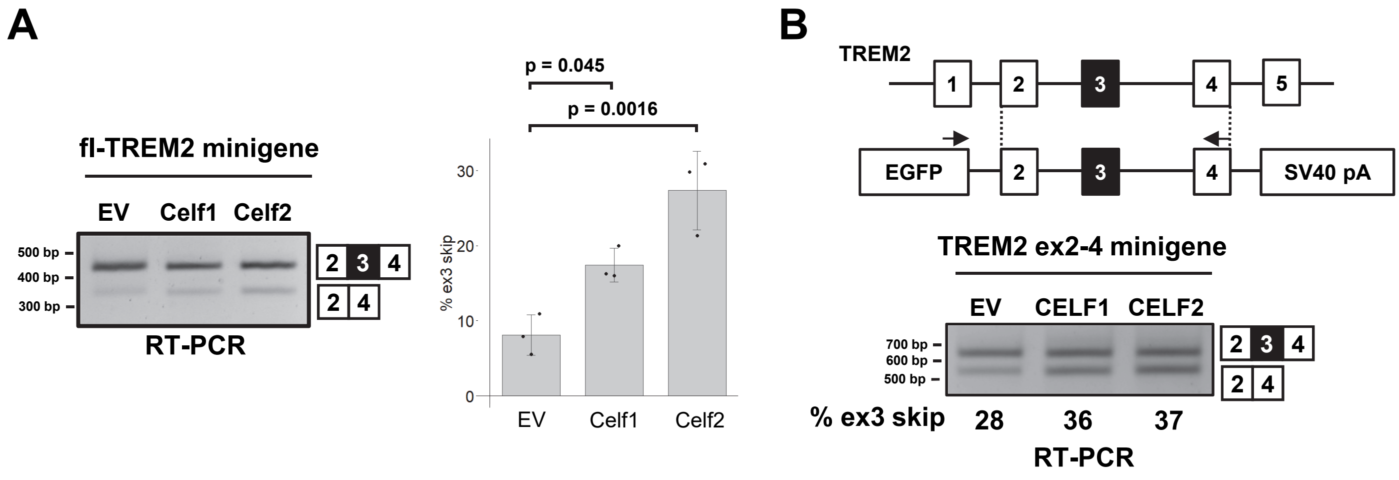

Figure S1. Effects of CELF proteins on TREM2 exon 3 splicing.

(**A**) Quantitative analysis of exon 3 skipping by mouse Celf1 and Celf2 using the fl-TREM2 minigene. RT-PCR products were resolved by agarose gels. The bar chart shows the portion of exon 3 skipping, and error bars represent SD (n = 3). Tukey's test was used for statistical evaluation.

(**B**) RT-PCR analysis of HEK cells that were transfected with TREM2 ex2-4 minigene and human CELF proteins. The arrows indicate the primer set used to detect the splicing pattern of TREM2 exon 3. RT-PCR products were resolved with agarose gels.

Figure. S2

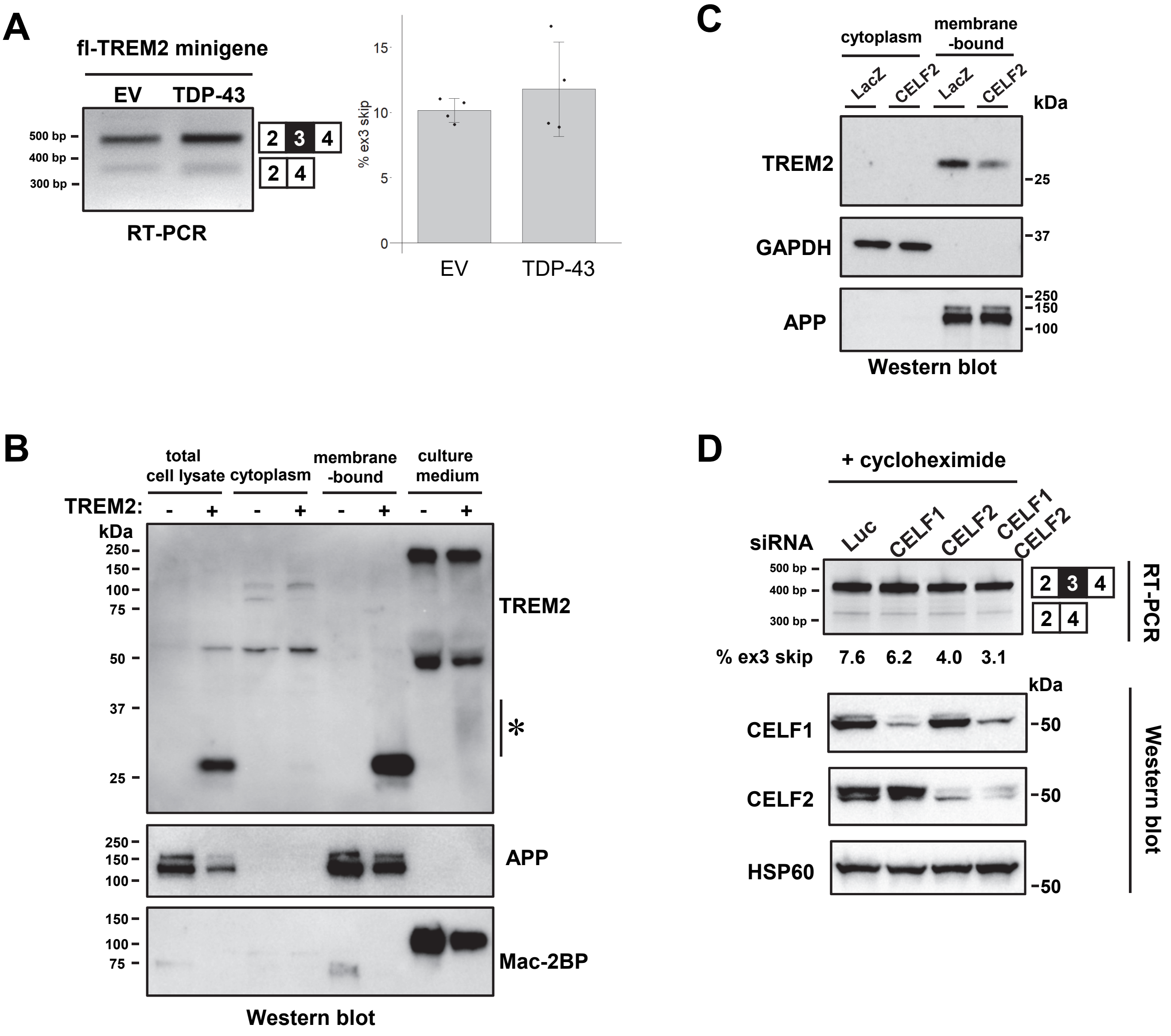

A
fl-TREM2 minigene
EV
TDP-43
500 bp
400 bp
300 bp
2 3 4
2 4
RT-PCR
% ex3 skip
15
10
5
0
EV
TDP-43
C
cytoplasm
membrane -bound
LacZ
CELF2
LacZ
CELF2
kDa
TREM2
GAPDH
APP
25
37
250
150
100
Western blot
B
total cell lysate
cytoplasm
membrane -bound
culture medium
TREM2:
-
+
-
+
-
+
-
+
kDa
250
150
100
75
50
37
25
TREM2
*
APP
Mac-2BP
Western blot
D
+ cycloheximide
siRNA
Luc
CELF1
CELF2
CELF1 CELF2
500 bp
400 bp
300 bp
2 3 4
2 4
RT-PCR
% ex3 skip
7.6
6.2
4.0
3.1
kDa
CELF1
CELF2
HSP60
50
Western blot

Figure S2. Splicing analyses of TREM2 in HEK cells.

(**A**) The TREM2 ex2-4 minigene was transfected with EGFP-fused human TDP-43 into HEK cells and the splicing pattern was detected by RT-PCR. The bar chart shows the portion of exon 3 skipping. Error bars represent SD (n = 4). TDP-43 induced no significant change in exon 3 skipping according to the two-tailed unpaired *t*-test.

(**B**) Western blot analysis of fractionated HEK cells with or without stable TREM2 expression. The cells were fractionated to obtain the membrane and cytoplasmic fractions. Proteins in the culture medium were concentrated through TCA precipitation and were detected using antibodies, as indicated. Although the TREM2 antibody bound to several bands (~28 kDa, 50 kDa, 100 kDa, 120 kDa, and 250 kDa), only the major band at ~28 kDa detected in the membrane-bound fraction was determined to be specific to TREM2-expressing cells. We noticed that a faint smear at approximately 30–40 kDa (indicated by *) was also detected in the TREM2-expressing cells and was likely to be the secreted soluble TREM2. APP and Mac-2BP are used as markers for membrane proteins and secreted proteins, respectively.

(**C**) The HEK cells were transfected with either EGFP-LacZ or EGFP-CELF2 and fractionated. The cytoplasmic and membrane fractions were subjected to western blot analysis, and it was determined that CELF2 overexpression reduced TREM2 in the membrane fraction.

(**D**) The upper panel shows the splicing pattern of TREM2 exon 3 in the fl-TREM2 minigene stable cell line treated with siRNA, as indicated. The RT-PCR products were resolved with polyacrylamide gels. Luc indicates siRNA targeting luciferase. The lower panel shows western blotting analysis to confirm gene silencing by siRNA treatments.

Figure. S3

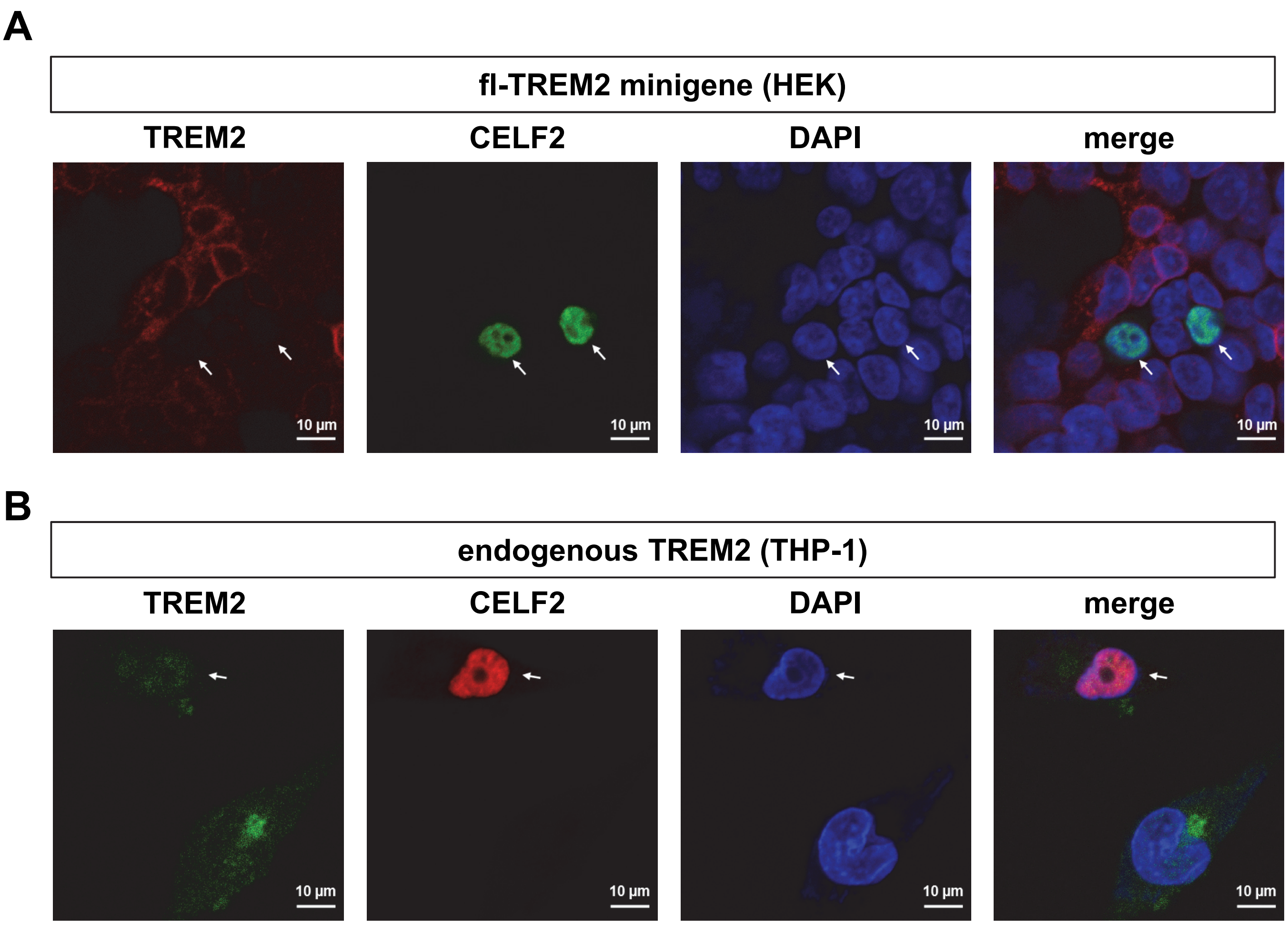

Figure S3. CELF2 reduces TREM2 protein expression.

(**A**) Immunofluorescence analysis of cells stably expressing the fl-TREM2 minigene using the anti-TREM2 antibody (red). Cell nuclei were stained with Hoechst dye (blue). Scale bar, 10 µm.

(**B**) Immunofluorescence analysis of endogenous TREM2 in THP-1 cells. The arrows indicate THP-1 cells expressing mCherry-fused CELF2 (red) introduced by electroporation. Cell nuclei were stained with Hoechst dye (blue). Scale bar, 10 µm.

Figure. S4

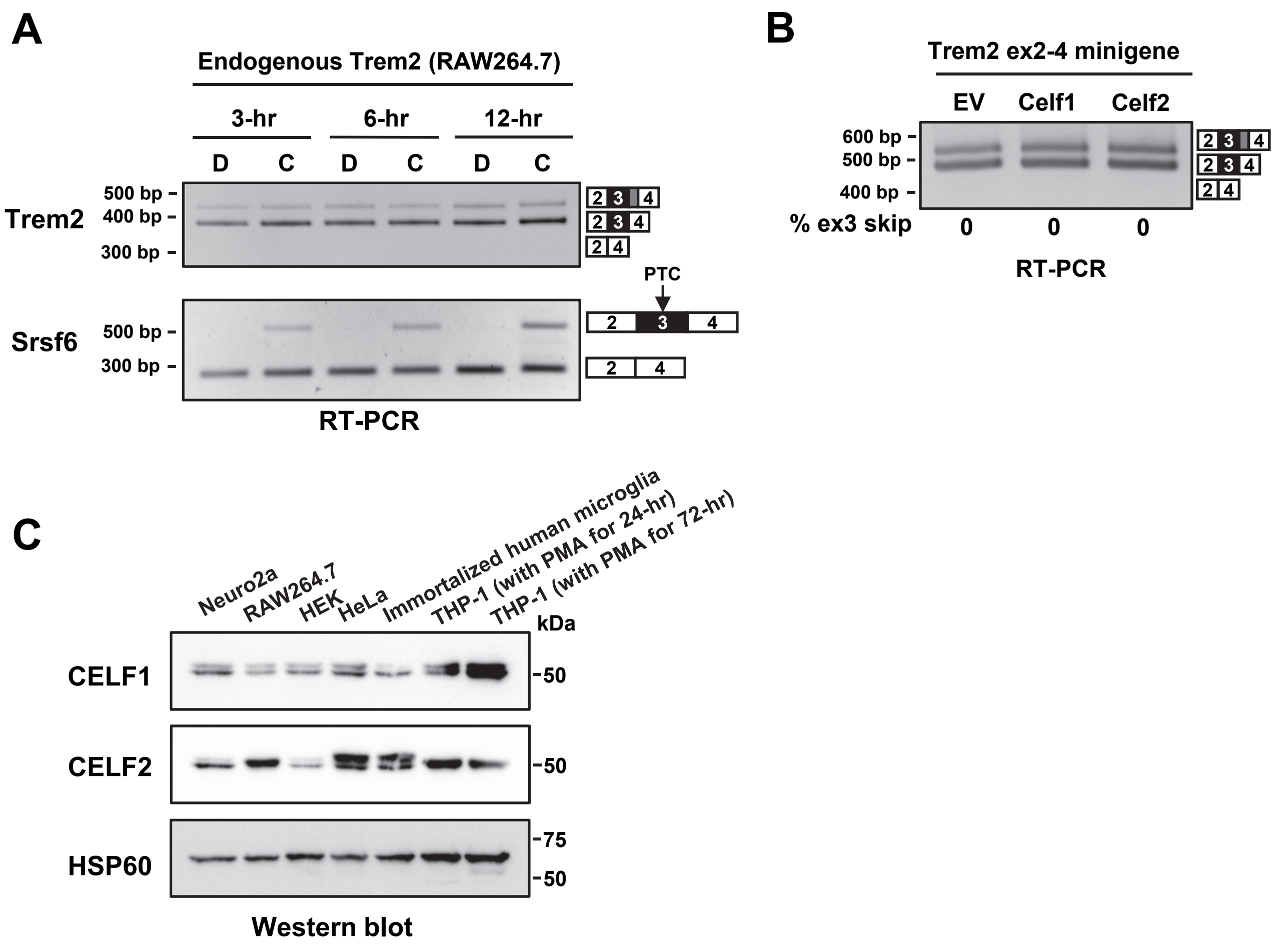

Figure S4. The species-specific splice pattern of exon 3 and CELF protein expression in different cell lines.

(**A**) RT-PCR analysis of RAW264.7 treated with CHX to inhibit NMD at different time points. Srsf6 was used as a positive control to confirm NMD inhibition [27]. A spliced product of Srsf6 containing exon 3 was undetectable due to degradation by NMD under basal conditions, but appeared on CHX treatment for 12 h. Trem2 did not show exon 3 skipping even after treatment with CHX for 12 h. PTC: premature termination codon. D and C indicate DMSO and CHX, respectively. RT-PCR products were resolved by polyacrylamide gels.

(**B**) Neuro2a cells were transfected simultaneously with MmTrem2 ex2-4 minigene and EGFP-fused mouse Celf1 or Celf2 constructs. The splicing patterns were detected by RT-PCR using agarose gels. No exon 3 skipping was observed.

(**C**) Western blot analyses of endogenous CELF proteins in mouse (Neuro2a and RAW264.7) and human (others) cell lines. HSP60 serves as a loading control.

Figure. S5

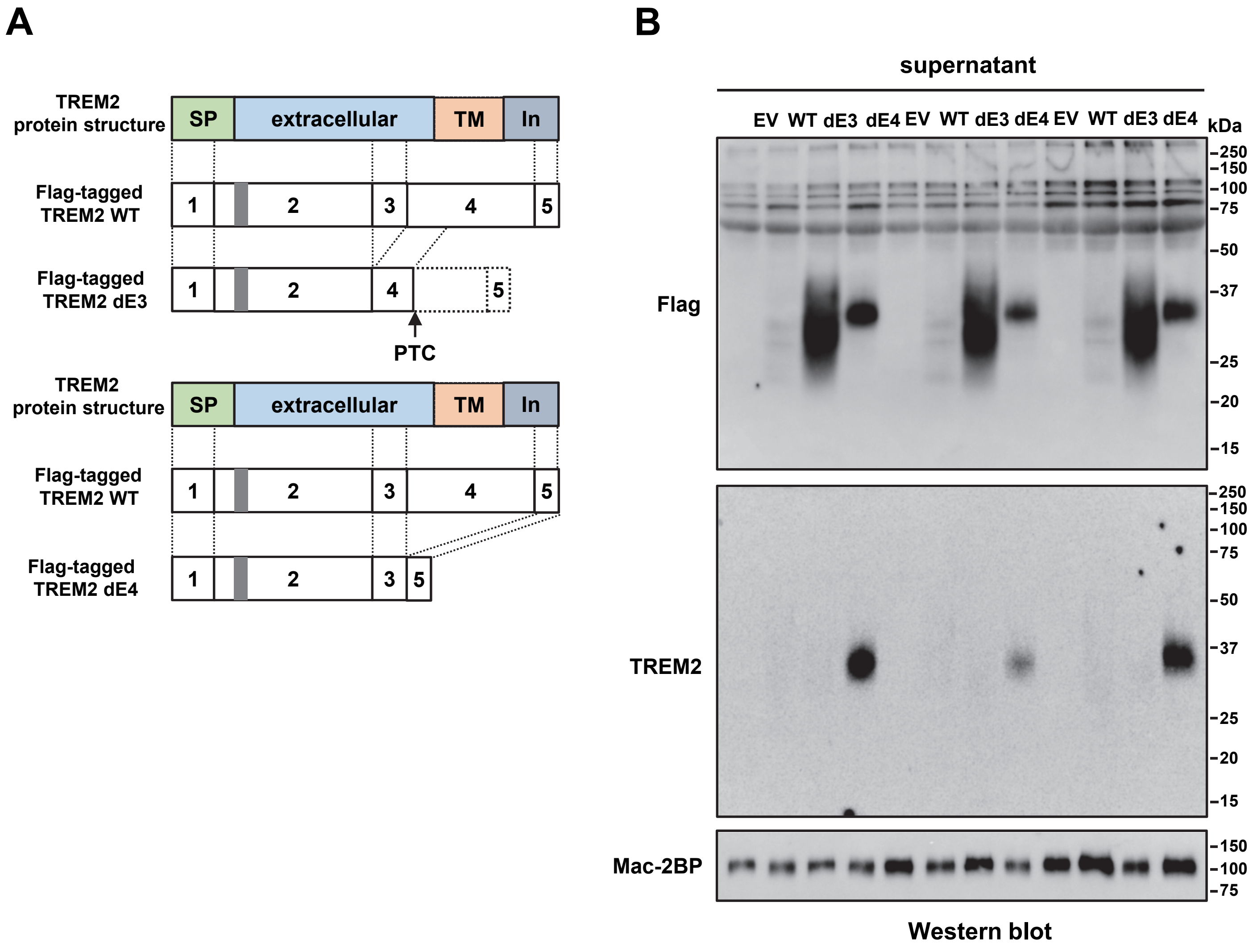

Figure S5. Secretion of TREM2 splice variants.

(**A**) Schematic diagrams of TREM2 splice variants (dE3 and dE4) and corresponding functional regions. These variants were inserted into the pCMV-FRT vector. The DDDDK tag indicated by a gray box was added just after the signal peptide. Exon 3 skipping in dE3 generates a PTC on exon 4, resulting in the loss of a transmembrane (TM) region. SP: signal peptide. In: intracellular region.

(**B**) The expression of the TREM2 variants in the culture medium of HEK cells. Secreted proteins in the culture medium were concentrated by TCA precipitation and resolved by SDS-PAGE. Secreted TREM2 variants were detected by western blotting using anti-TREM2 and anti-DDDDK-tag antibodies. EV represents the pCMV-FRT empty vector. Mac-2BP served as a loading control.

Figure. S6

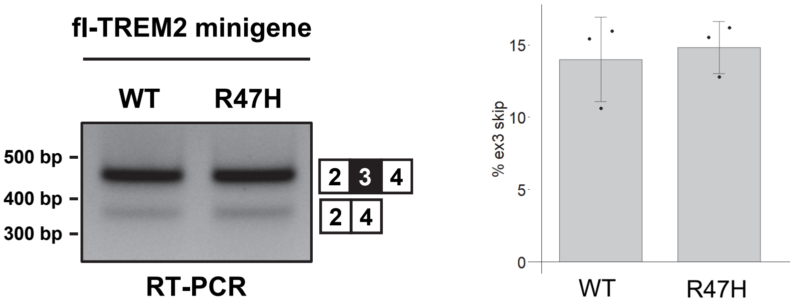

Figure S6. The effects of the R47H substitution on the splicing of TREM2 exon 3.

The splicing patterns of WT and R47H exon 3. Each minigene was transfected into HEK cells. The primer set is the same as in Figure 1A. RT-PCR products were resolved by an agarose gel. Error bars represent SD (n = 3). No significant alteration was observed (two-tailed unpaired *t*-test).

Figure. S7

Fig. 1B

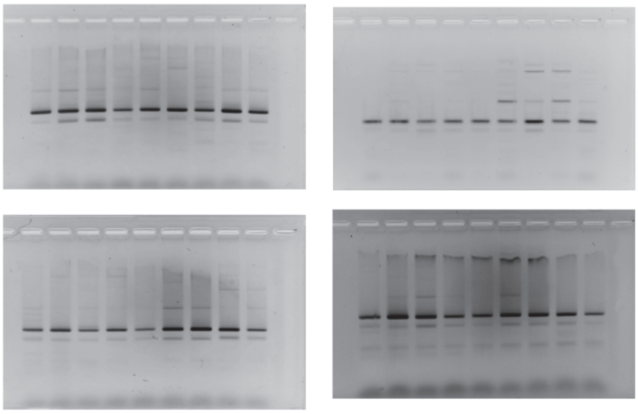

Fig. 1C

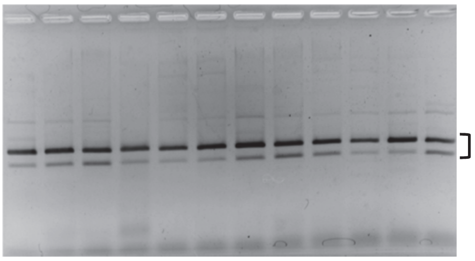

Fig. 2A

GFP

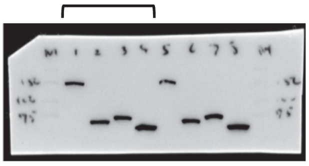

Lamin B

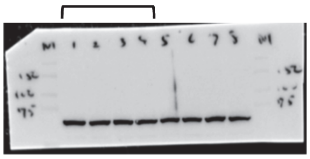

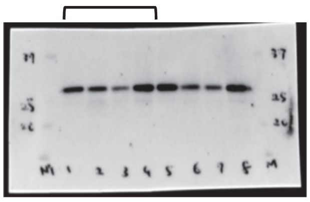

TREM2

Fig. 2B

GFP

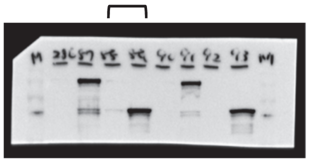

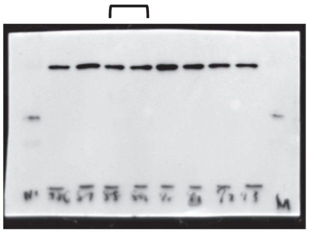

GAPDH

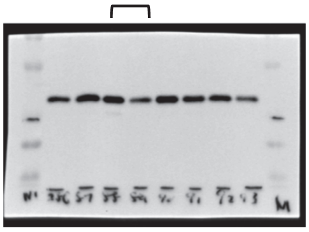

TREM2

Fig. 2C

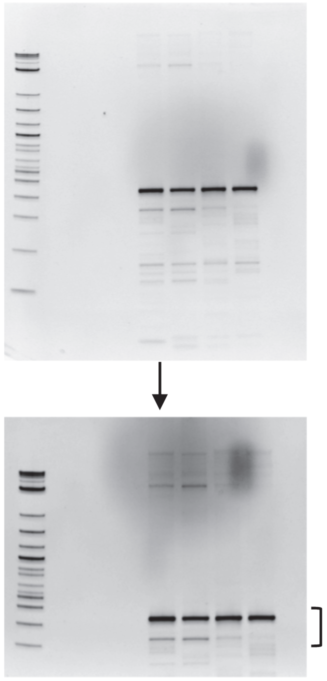

CELF1

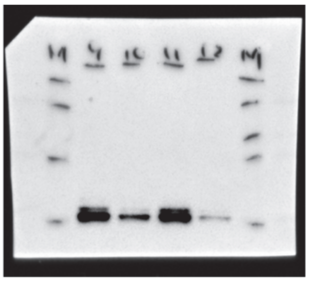

CELF2

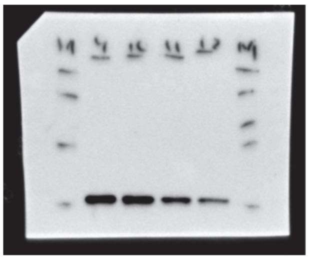

Lamin B

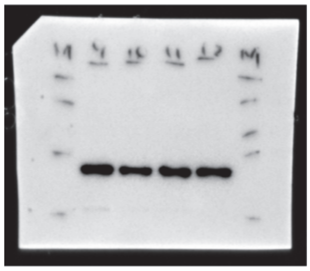

Fig. 3A

GFP

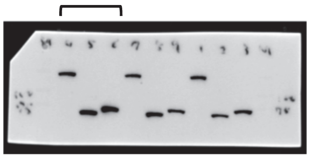

Lamin B

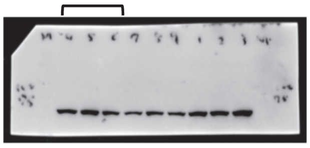

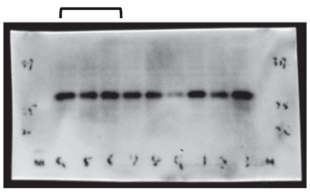

TREM2

Fig. 3B

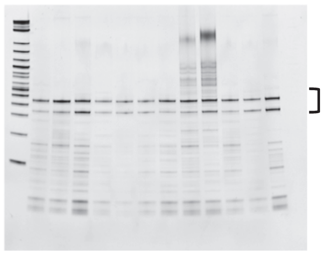

Fig. 3C

GFP

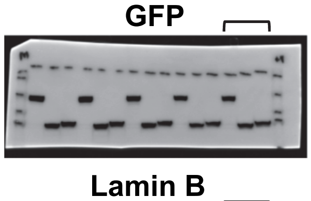

Lamin B

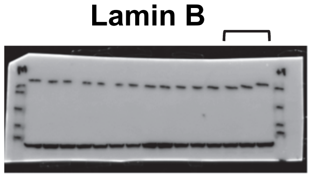

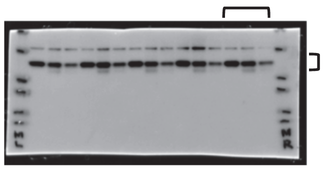

TREM2

Figure. S7

Fig. 4A

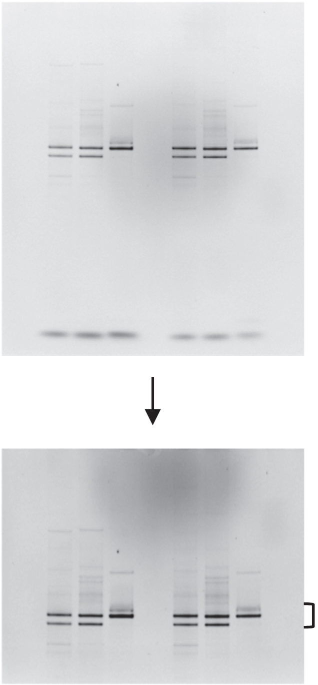

Fig. 4C

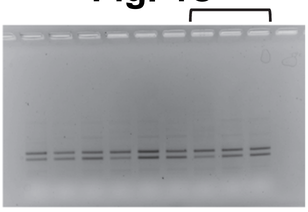

Hs

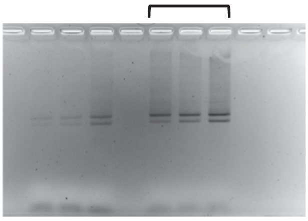

Cs

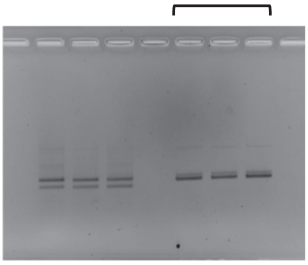

Mm

Fig. 5A

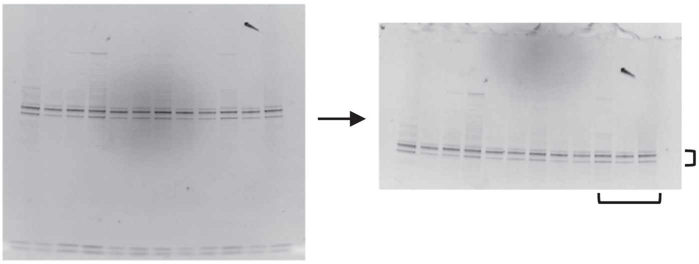

Fig. 5B

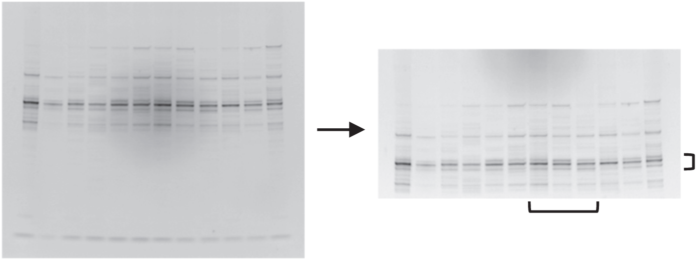

Fig. 5C

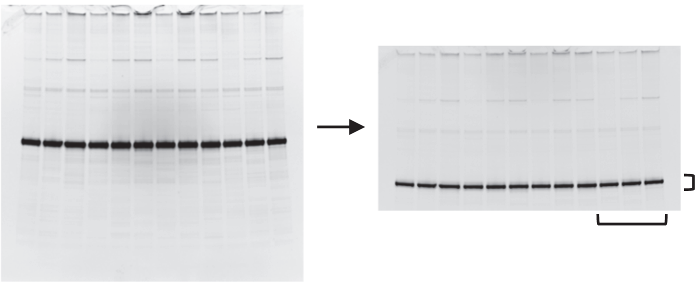

Fig. 5D

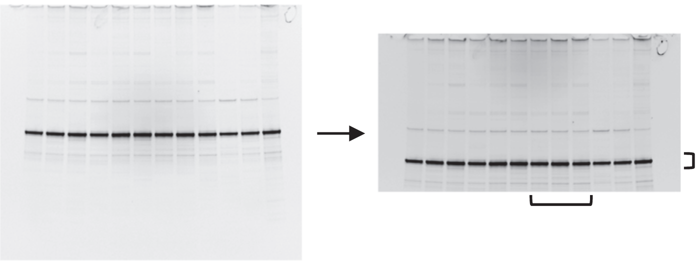

Fig. 5E

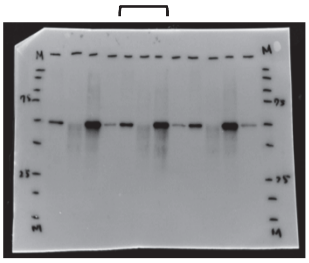

CELF2

Fig. 5F

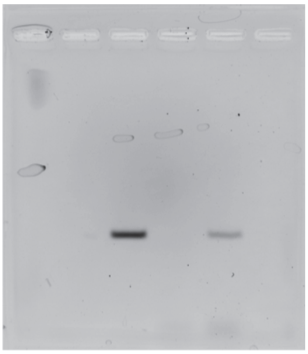

Figure. S7

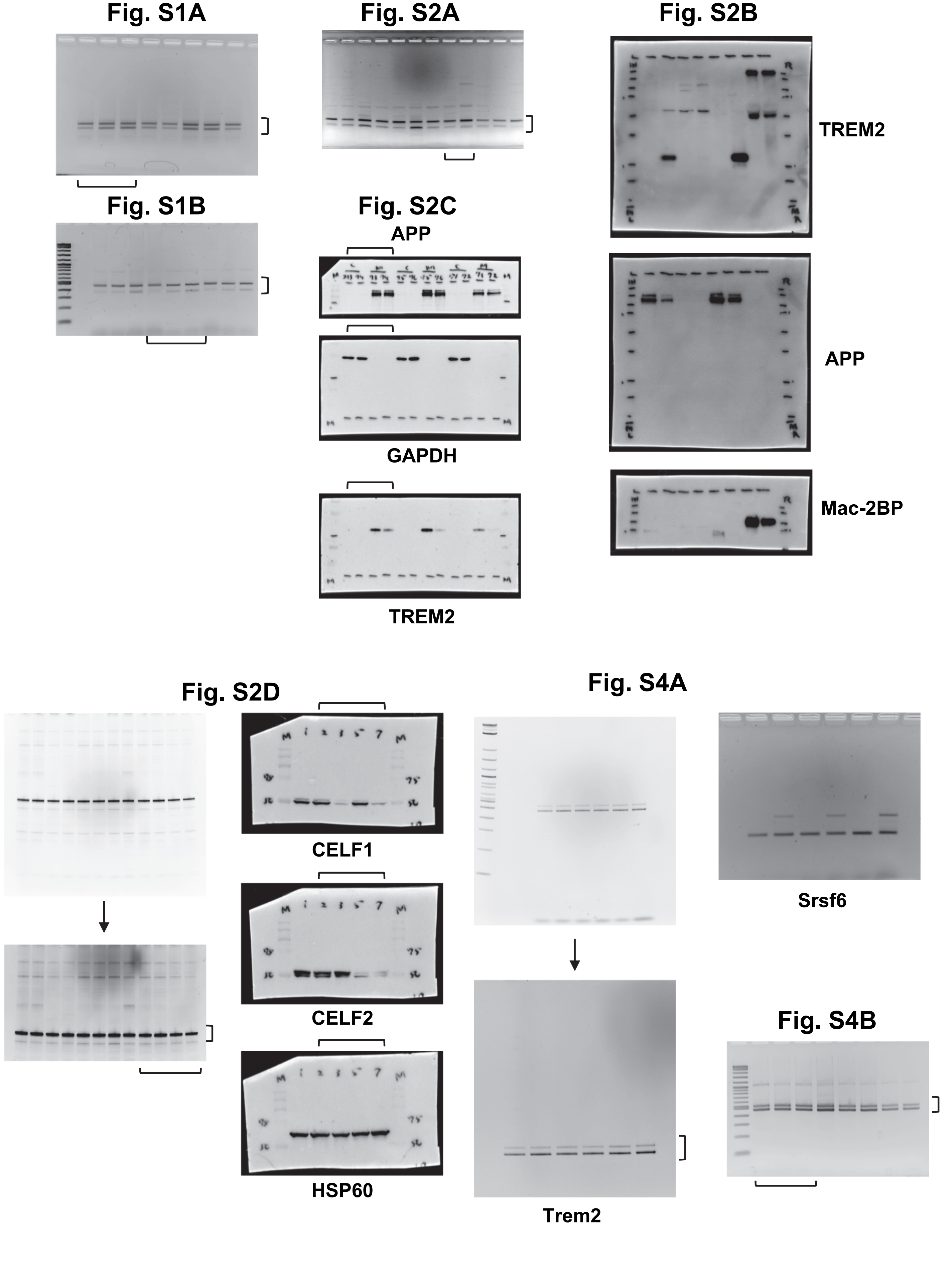
Fig. S1A
Fig. S2A
Fig. S2B
TREM2
Fig. S1B
Fig. S2C
APP
GAPDH
TREM2
APP
Mac-2BP
Fig. S2D
CELF1
CELF2
HSP60
Fig. S4A
Srsf6
Trem2
Fig. S4B

Figure. S7

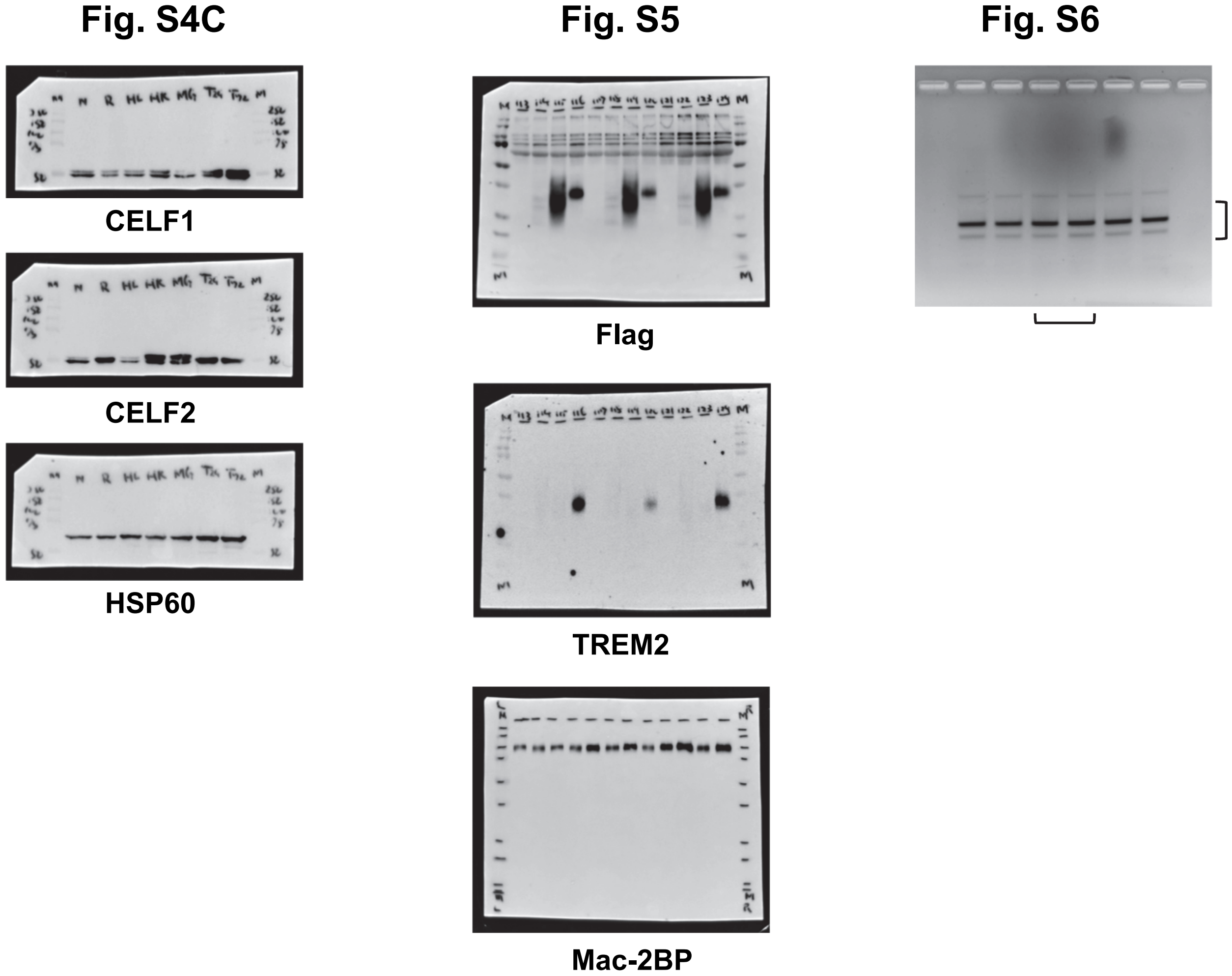

Figure S7. Original images of western blot and RT-PCR analyses.

For some RT-PCR results, we included two images: one used for analysis, and the other for showing a larger gel area.
